# Transglutaminase 2 in human peritoneal dialysis‐related peritoneal injury

**DOI:** 10.14814/phy2.70567

**Published:** 2025-09-22

**Authors:** Shunnosuke Kunoki, Masashi Ikeno, Hideki Tatsukawa, Yukinao Sakai, Hiroshi Kinashi, Keisuke Kamiya, Masafumi Suzuki, Masashi Mizuno, Makoto Yamaguchi, Hiroyuki Sasakura, Yuki Morioka, Masataka Banshodani, Mitsuhiro Tawada, Masato Iwabu, Takuji Ishimoto, Kosei Takeuchi, Kiyotaka Hitomi, Hideki Kawanishi, Yasuhiko Ito

**Affiliations:** ^1^ Department of Nephrology and Rheumatology Aichi Medical University Nagakute Aichi Japan; ^2^ Department of Endocrinology Metabolism and Nephrology, Nippon Medical School Tokyo Japan; ^3^ Department of Medical Cell Biology Aichi Medical University Nagakute Aichi Japan; ^4^ Cellular Biochemistry Lab, Graduate School of Pharmaceutical Sciences Nagoya University Nagoya Japan; ^5^ Department of Nephrology Nagoya University Nagoya Japan; ^6^ Department of Surgery and Artificial Organs, Akane‐Foundation Tsuchiya General Hospital Hiroshima Japan; ^7^ Department of Nephrology Imaike Jin Clinic Nagoya Nagoya Japan

**Keywords:** α‐SMA, collagen density, peritoneal fibrosis, TG2‐TGF‐β1 interaction, transglutaminase 2

## Abstract

Patients undergoing long‐term peritoneal dialysis (PD) frequently develop peritoneal fibrosis and angiogenesis, leading to membrane dysfunction. Transglutaminase 2 (TG2) stabilizes the extracellular matrix against proteases. In an animal model, inhibition of TG2 reduced peritoneal fibrosis, angiogenesis, and inflammation. We investigated the expression of TG2 in 163 human peritoneal membrane tissue samples, including controls, tissues exposed to conventional acidic or low‐glucose degradation product (GDP) pH‐neutral solutions, and those with peritonitis or encapsulating peritoneal sclerosis (EPS), and explored the role of TG2 in high‐glucose–induced pathophysiology in mesothelial cells. TG2 expression was upregulated in association with peritoneal membrane injury and was the highest in peritonitis. TG2 expression was correlated with peritoneal membrane thickness, CD68‐positive macrophages, and myofibroblast expression. TG2 was expressed in mesothelial cells, α‐smooth muscle actin‐positive myofibroblast expression, macrophages, and endothelial cells in the diseased state. In cultured mesothelial cells, high‐glucose–induced upregulation of collagen 1, TGF‐β1, and TG2 was suppressed by a TG2 inhibitor or by TGF‐β1 small interfering RNA. TG2 is involved in the development of peritoneal injury during PD. High‐glucose dialysate is involved in the induction of peritoneal fibrosis through the interactive regulation of TGF‐β and TG2. Targeting TG2 may offer therapeutic potential for managing PD complications and EPS.

## 1 INTRODUCTION

In patients undergoing long‐term peritoneal dialysis (PD), peritoneal fibrosis and angiogenesis develop in association with peritoneal membrane dysfunction, which is a major cause of withdrawal from PD. Encapsulating peritoneal sclerosis (EPS) is a rare but serious and life‐threatening complication of PD. During the development of simple peritoneal fibrosis, a second hit, such as frequent peritonitis, induces EPS (Ito et al., 2024; Moinuddin et al., 2014). High collagen density in peritoneal fibrosis is reportedly associated with the development of EPS (Morelle et al., 2015; Tawada et al., 2016). We had recently reported that in all studies comparing pathology between conventional acidic and low‐glucose degradation product (GDP) pH‐neutral solutions, vasculopathy (L/V ratio: the ratio of luminal diameter [L] to vessel diameter [V]) dramatically improved in the low‐GDP pH‐neutral solution groups (Tawada et al., 2016). However, the extent of fibrosis was similar or only slightly improved in the low‐GDP pH‐neutral solution groups (Ito et al., 2024), indicating that glucose toxicity in the PD dialysate may be an important factor in the development of peritoneal fibrosis.

Transglutaminase 2 (TG2) has been reported to be a stabilizer of the extracellular matrix against proteases (Collighan & Griffin, 2009; Fell et al., 2021; Niger et al., 2013; Philp et al., 2018) through a matrix cross‐linking process that is crucial for the activation of transforming growth factor (TGF)‐β (Burhan et al., 2016; Nunes et al., 1997). In addition, TG2 is involved in cell survival via the cell adhesion process (Iismaa et al., 2009; Nadalutti et al., 2011). Thus, TG2 contributes to disease progression in fibrosis and cancer (Collighan & Griffin, 2009; Soltani & Kaartinen, 2023; Tatsukawa & Hitomi, 2021), suggesting that TG2 is a key enzyme in the pathogenesis of peritoneal fibrosis and increases collagen density during PD treatment. We recently reported that TG2 is involved in the development of peritoneal injury and fibrosis in a model of chlorhexidine gluconate (CG)‐induced peritoneal fibrosis (Kunoki et al., 2023). Genetic deletion of TG2 reduced TGF‐β expression, peritoneal fibrosis, and angiogenesis (Kunoki et al., 2023). Moreover, treatment with a TGF‐β receptor inhibitor reduced the peritoneal fibrosis associated with the reduction of TG2 expression, indicating an interaction between TGF‐β and TG2.

The structure of the human peritoneal membrane and fibrosis are significantly different from those of rodents (Ito et al., 2024). In addition, accurate animal models of EPS in human peritoneal pathology are currently lacking (Ito et al., 2024; Nakayama et al., 2023). Therefore, studies using biopsied human peritoneal tissues are important to evaluate the mechanisms and status of human PD‐related peritoneal injury (Ito et al., 2024). The present study investigated the expression of TG2 in the human peritoneal membrane after PD treatment and EPS, and explored the role of TG2 in high‐glucose–induced pathophysiology in mesothelial cells.

## 2 MATERIALS AND METHODS

### 2.1 Human studies

Human peritoneal tissue experiments were performed in accordance with the Declaration of Helsinki and approved by the Human Research Ethics Committee of the Faculty of Medicine, Nagoya University (Nagoya, Japan; approval number 299‐7) and the Human Research Ethics Committee of Tsuchiya General Hospital (Hiroshima, Japan; approval number E160530‐1). Written informed consent was obtained from all study participants.

### 2.2 Histological and immunohistochemical assessments

**FIGURE 1:**
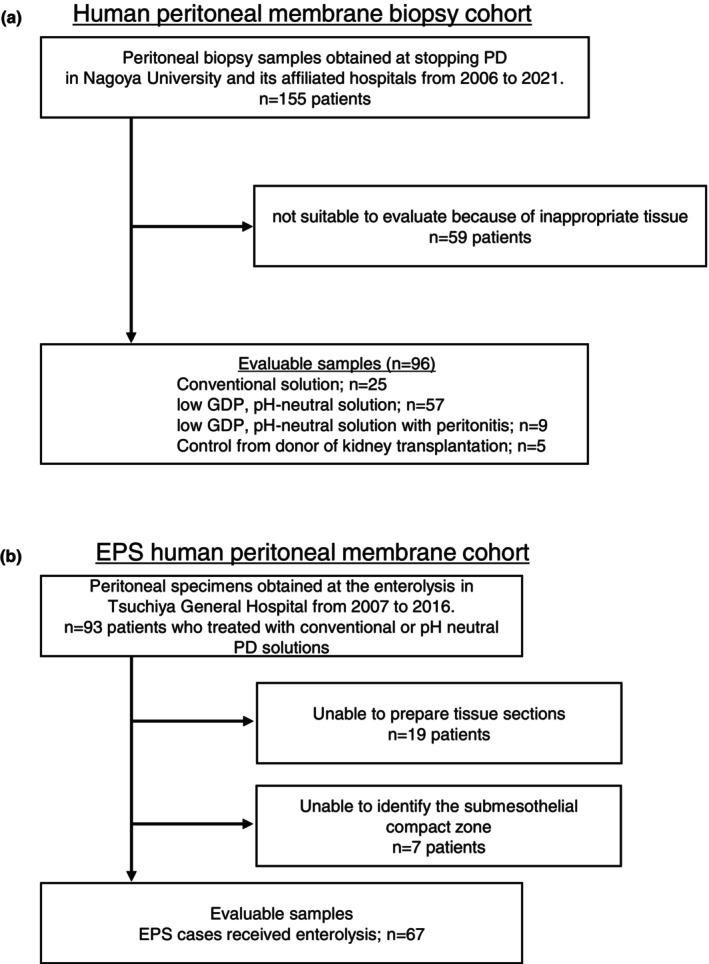
Flow diagram of the study population. (a) Human peritoneal membrane biopsy cohort. (b) EPS human peritoneal membrane cohort.

Human peritoneal membrane tissues were obtained from the cohort of patients who underwent peritoneal membrane biopsy during catheter removal for PD cessation at Nagoya University and its affiliated hospitals. Parietal peritoneal tissues from the EPS human peritoneal membrane cohort were obtained during surgical enterolysis at Tsuchiya General Hospital (Figure 1). All participants were Japanese and aged over 18 years. Peritoneal tissues of approximately 1 cm × 1 cm were obtained from the anterior abdominal wall at the time of PD catheter removal for patients with PD or at the time of kidney transplantation for controls, as previously described (Kinashi et al., 2013; Kinashi et al., 2019; Sugiyama et al., 2021; Tawada et al., 2021). The peritoneal membrane tissues were fixed using Maskedform (Japan Tanner, Osaka, Japan), a formalin fixative, and embedded in paraffin. Four‐micrometer‐thick sections were deparaffinized, rehydrated, and stained with hematoxylin and eosin (HE) or Masson's trichrome using a routine method. To evaluate peritoneal thickening, the submesothelial compact zone was identified, and the thickness was measured at five points for each peritoneal tissue. The mean of these measurements was calculated and defined as peritoneal thickness, as described previously (Sugiyama et al., 2021; Tawada et al., 2016; Tawada et al., 2021). The antibodies used for immunohistochemistry are listed in Table 1. Immunostaining for CD68, CD31, α‐smooth muscle actin (α‐SMA), and TG2 was performed on the paraffin‐embedded tissues. Sections were deparaffinized, rehydrated, and incubated with 3% hydrogen peroxide in methanol for 30 min to block endogenous peroxidase activity. For TG2 and CD68 staining, antigen retrieval was performed at 98°C for 30 min in citric acid solution prior to endogenous peroxidase blocking. Sections were then incubated with 10% normal goat serum (Nichirei Bioscience, Tokyo, Japan) for 30 min to block nonspecific binding. The sections were then incubated with anti‐human CD68 antibody (Dako, Glostrup, Denmark), anti‐mouse/rat/human α‐SMA antibody (Abcam, Cambridge, UK), or anti‐mouse/human TG2 antibody (Abcam), followed by incubation with a secondary antibody of horseradish peroxidase‐conjugated polymer (Nichirei Bioscience). Enzyme activity was detected using 3,3′‐diaminobenzidine (DAB) (Nichirei Bioscience). To analyze positively stained areas, at least five random independent fields of the sections were photographed at ×200 magnification using a microscope (BX51; Olympus, Tokyo, Japan). Positive areas were evaluated using image analysis software (ImageJ; Wayne Rasband (NIH), Bethesda, MA, USA). The number of CD‐68‐positive cells was counted as described previously (Kamiya et al., 2024; Sugiyama et al., 2021; Tawada et al., 2016; Tawada et al., 2021).

**TABLE 1:** List of antibodies used in this study.

Primary antibody	Catalogue ID	Company
Rabbit anti‐αSMA antibody	ab5694	Abcam, Cambridge, UK
Mouse anti‐human HBME‐1 antibody	M3505	Dako, Santa Clara, CA
Rabbit anti‐TGF‐β1 antibody	21898‐1‐AP	Proteintech, Chicago, IL
Mouse anti‐human CD31 antibody	M082329	Dako, Santa Clara, CA
Mouse anti‐human CD68 antibody	M087629	Dako, Santa Clara, CA
Mouse anti‐mouse/human TG2 antibody	ab2386	Abcam, Cambridge, UK
Secondary Antibody	Catalogue ID	Company
Goat anti‐mouse IgG antibody, HRP conjugate	424131	Nichirei Bioscience, Tokyo, Japan
Goat anti‐rabbit IgG antibody, HRP conjugate	424141	Nichirei Bioscience, Tokyo, Japan
Alexa 555 labeled goat anti‐mouse IgG	ab150114	Abcam, Cambridge, UK
Alexa 488 labeled goat anti‐rabbit IgG	A‐11070	Invitrogen, Waltham, MA
FITC labeled rabbit anti‐mouse IgG	F0261	Dako Glostrup, Denmark
FITC labeled goat anti‐mouse IgM	1020‐02	SouthernBiotech, Birmingham, AL

### 2.3 Double immunofluorescence staining

To identify the localization of TG2 expression in peritoneal tissues, double immunofluorescence staining was performed on human peritoneal tissue samples, as described previously (Kunoki et al., 2023; Sugiyama et al., 2021). For double staining of TG2 and CD68, CD31, α‐SMA, or HBME‐1, 4‐μm‐thick paraffin sections were deparaffinized and rehydrated. Antigen retrieval procedures were performed with citric acid solution (pH 6.0, Immuno Active IA‐6500; Matsunami Glass, Osaka, Japan) at 90°C for 30 min, after which the sections were incubated with 1% normal bovine serum albumin (BSA) in phosphate‐buffered saline (PBS) for 30 min. The sections were then incubated with a mixture of rabbit anti‐TG2 antibody and anti‐HBME‐1, anti‐human CD31 antibody (Dako), anti‐CD68, or anti‐α‐SMA antibody overnight at 4°C. After washing with PBS, the sections were incubated with the secondary antibodies Alexa Fluor 555‐conjugated goat anti‐mouse IgG or Alexa Fluor 488‐conjugated goat anti‐rabbit IgG and fluorescein isothiocyanate (FITC)‐conjugated rabbit anti‐mouse IgG or FITC‐conjugated goat anti‐mouse IgM for 30 min. Positive signals were observed using confocal microscopy (FV‐3000; Olympus, Tokyo, Japan) equipped with objective lenses (UPLXAPO10X and UPLXAPO40X; Olympus) and recorded with FV31S‐SW software (Olympus).

### 2.4 Cell culture study

The human mesothelial cell line Met‐5A (CRL‐9444; American Type Culture Collection [ATCC], Manassas, VA) was purchased and maintained according to ATCC guidelines as described previously (Kariya et al., 2018; Kinashi et al., 2013; Mizutani et al., 2010; Sun et al., 2019).

Met‐5A cells were cultured in serum‐free medium for 24 h. Silencer‐select small interfering RNA (siRNA) for TGF‐β (Hs01‐0010865, Sigma‐Aldrich, Ichikawa, Japan) and siRNA Universal Negative Control #1 (as control siRNA, Sigma‐Aldrich, Tokyo, Japan) were transfected into Met‐5A cells using Lipofectamine RNAiMAX Reagent (Thermo Fisher Scientific, Waltham, MA) according to the manufacturer's instructions. Transfection was performed for 24 h, after which the cells were harvested by incubation in a medium containing 5 or 90 mM glucose for 24 h. RNA was extracted from cell lysates. In another experiment to further investigate the relationship between TG2 and TGF‐β1, TG2 inhibitor (Zedira, GmbH, Darmstadt, Germany) (Kazemi‐Esfarjani & La Spada, 2010; Verhaar et al., 2011) was added to the medium containing 5 mM or 90 mM glucose to block TG2. Cells were harvested after 24 h of incubation, and RNA was extracted.

### 2.5 RNA isolation and reverse transcription‐polymerase chain reaction

Total RNA was extracted from cultured mesothelial cells using an RNeasy Mini Kit (Qiagen, Hilden, Germany), as described previously (Kariya et al., 2018; Sun et al., 2019; Tanaka et al., 2022; Terabayashi et al., 2015). One microgram of RNA was used to synthesize complementary DNA (cDNA) using a high‐capacity cDNA reverse transcription kit (Thermo Fisher Scientific, Waltham, MA, USA) according to the manufacturer's instructions. Quantitative real‐time polymerase chain reaction (PCR) was conducted using a StepOnePlus system (Applied Biosystems, Foster City, CA, USA), as described previously (Kariya et al., 2018; Sun et al., 2019; Tanaka et al., 2022; Terabayashi et al., 2015). The TaqMan gene expression assays (Applied Biosystems) used in this study are shown in Table S1. Glyceraldehyde‐3‐phosphate dehydrogenase was used as an internal standard. The company and catalogue numbers of the reagents used are listed in Table S2.

### 2.6 Statistical analyses

Statistical analyses were performed using SPSS Statistics version 26.0 (IBM, Chicago, IL). The normality of distributions was assessed using the Shapiro–Wilk test. To test for differences between groups, Student's *t*‐test or Mann–Whitney *U* test was used to compare normally distributed and nonparametric data, respectively. For groups of three or more, a one‐way analysis of variance followed by Tukey's test was used. Differences were considered significant when *p*‐values were less than 0.05.

## 3 RESULTS

### 3.1 Expression of TG2 and markers of peritoneal membrane injury

A total of 163 peritoneal membrane tissues, including control samples (*n* = 5); tissues obtained after treatment with low‐GDP pH‐neutral PD solutions (*n* = 57) and conventional acidic PD solutions (*n* = 25); and tissues showing peritonitis (*n* = 9) and EPS (*n* = 67) were examined (Figure 1). The patient characteristics of the human peritoneal membrane biopsy cohort are summarized in Table 2. The incidence rate of PD‐related peritonitis was 0.29 ± 0.49 episodes per patient‐year in the low‐GDP pH‐neutral PD solution group and 0.30 ± 0.32 in the conventional acidic PD solution group. The characteristics of the EPS cohort were as follows: mean age, 55.7 ± 10.1 years; the underlying cause of renal failure was chronic glomerulonephritis in 92.8% of the cases; the duration of PD treatment was 128.1 (22, 235) (median, interquartile range [IQR]) months; and the incidence rate of peritonitis was 0.27 ± 0.47 episodes per patient‐year.

**TABLE 2:** Patient characteristics in the human peritoneal membrane biopsy cohort.

	Neutral solution	Conventional solution	Peritonitis
	(*n* = 57)	(*n* = 25)	(*n* = 9)
Age (year)	61.5 ± 12.4	55.0 ± 8.6	58.9 ± 12.1
Male, *n* (%)	80%	48%	75.0%
Primary kidney disease			
Chronic glomerulonephritis, *n* (%)	37.5%	52.0%	0%
Diabetes nephropathy, *n* (%)	35.7%	16.0%	50.0%
Others, *n* (%)	26.8%	32.0%	50.0%
PD duration (months)	43 (17–72)	92 (67–113)	17 (8–28)
Peritonitis rate (episodes per patient‐year)	0.29 ± 0.49	0.30 ± 0.32	1.13 ± 0.88
D/P Cr	0.71 ± 0.16	0.70 ± 0.15	0.68 ± 0.17^a^
Use of icodextrin, *n* (%)	71.4%	52.0%	50.0%
Reason for withdrawal from PD			
Dialysis failure/UF failure, *n* (%)	28.6%	48.0%	0.0%
Prevention of EPS, *n* (%)	19.6%	40.0%	0.0%
Peritonitis *n* (%)	0%	0%	100.0%

Abbreviations: conventional solution, conventional acidic solution; D/P Cr, dialysate‐to‐plasma ratio of creatinine; EPS, encapsulating peritoneal sclerosis; Neutral solution, low‐GDP pH‐neutral solution; UF, ultrafiltration.

^a^ D/P Cr of the peritonitis group was measured before peritonitis.

**FIGURE 2:**
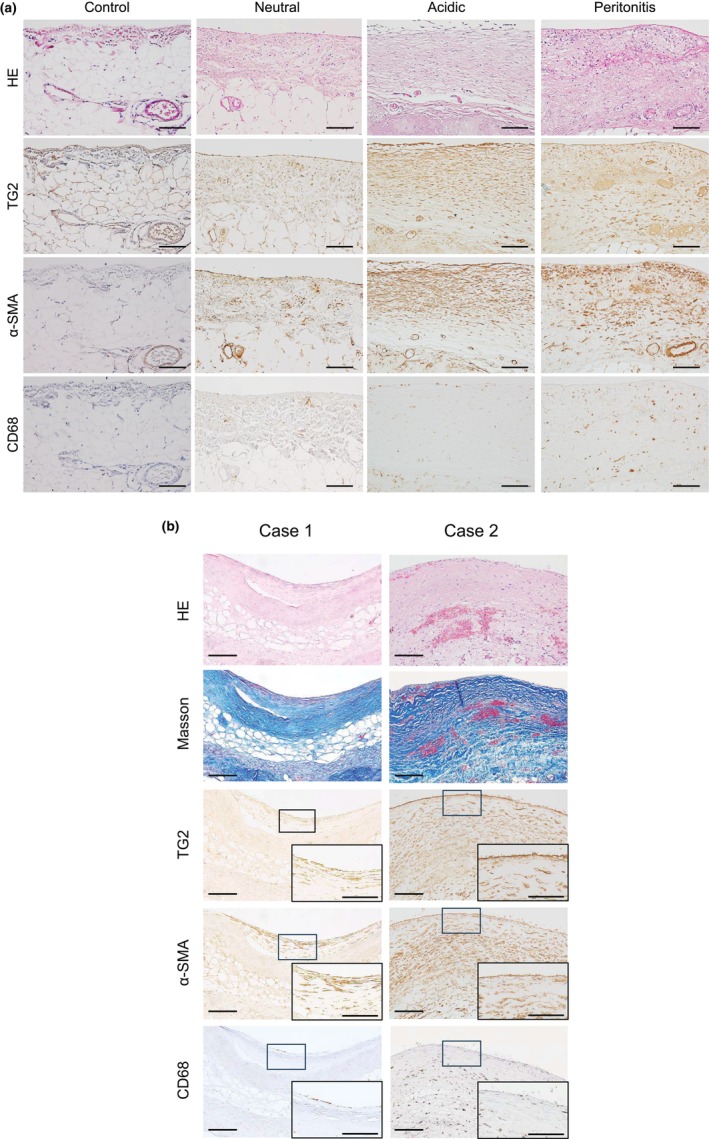
TG2 expression in human peritoneal membranes. (a) Representative images of human peritoneal membrane staining in the control group; after treatment with low‐GDP pH‐neutral PD solutions (Neutral) and conventional acidic PD solutions (Acidic); and in patients with peritonitis. α‐SMA, α‐smooth muscle actin; CD68, CD68‐positive cells; HE, Hematoxylin and eosin; TG2, Transglutaminase 2. (b) Representative images of human EPS cases: Case 1: Significant sclerotic changes with a decrease in the cell number. TG2 and α‐SMA‐positive myofibroblasts were observed in the newly synthesized encapsulating membrane. Case 2: Strong expression of TG2 and α‐SMA associated with intraperitoneal bleeding. However, inflammatory changes were also observed. Scale bars = 100 μm. Inset. The right lower squares in the figures represent enlargements of the small squares. Scale bars in the small square column = 50 μm.

**FIGURE 3:**
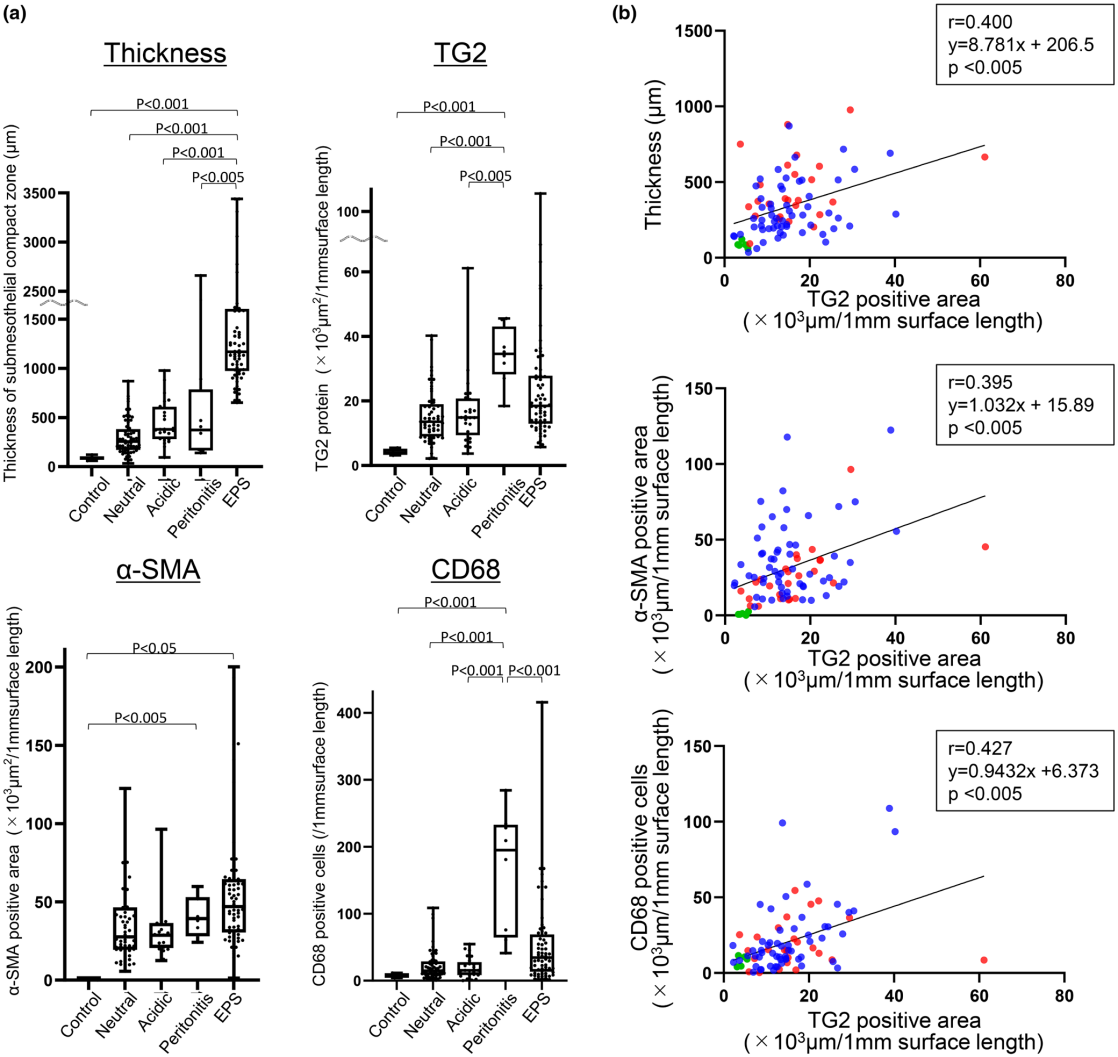
Analyses of peritoneal thickness and TG2, α‐SMA, and CD68 expression in human peritoneal membranes. (a) Quantitative analysis and comparison of peritoneal wall thickness, TG2, α‐SMA, and CD68 expression in the control peritoneum; the peritoneal membrane after treatment with low‐GDP pH‐neutral solutions and conventional acidic solutions, peritonitis; and the membrane showing EPS. The boxes indicate the interquartile range, and the whiskers reflect within 1.5 times the interquartile range. (b) TG2 expression showed correlations with peritoneal membrane thickness, α‐SMA expression, and the number of CD68‐positive cells. α‐SMA, α‐smooth muscle actin; Acidic, Conventional acidic solution group; Blue circle, Case treated with low‐GDP pH‐neutral solution; EPS, Encapsulating peritoneal sclerosis; Green circle, Control peritoneal membrane; Neutral, Low‐GDP pH‐neutral solution group; Red circle, Case treated with conventional acidic solution; TG2, Transglutaminase 2.

We compared the peritoneal thickness and expression of TG2, α‐SMA, and CD68 in the control group, patients treated with low‐GDP pH‐neutral solution, patients treated with conventional acidic solution, patients with peritonitis, and those with EPS. The thickness of the peritoneal membrane in the control group was 85.1 (81.4–86.7) (median, IQR) μm. Peritoneal thickness was the greatest in patients with EPS (Figures 2a,b and 3a). Weak TG2 expression was detected in mesothelial cells. TG2 expression was upregulated in diseased conditions and was the highest in patients with peritonitis (Figures 2a,b and 3a). Alpha‐SMA expression was higher in EPS and peritonitis than in control peritoneal membranes (Figures 2a,b and 3a). CD68‐positive macrophages were significantly greater in peritonitis and EPS cases, with the highest increase observed in patients with peritonitis (Figures 2a,b and 3a).

TG2 expression was correlated with peritoneal thickness, α‐SMA‐positive myofibroblasts, and the expression of CD68‐positive macrophages (Figure 3b), as observed in our previously reported CG model (Kunoki et al., 2023).

### 3.2 Localization of TG2 expression in control and diseased human peritoneum

**FIGURE 4:**
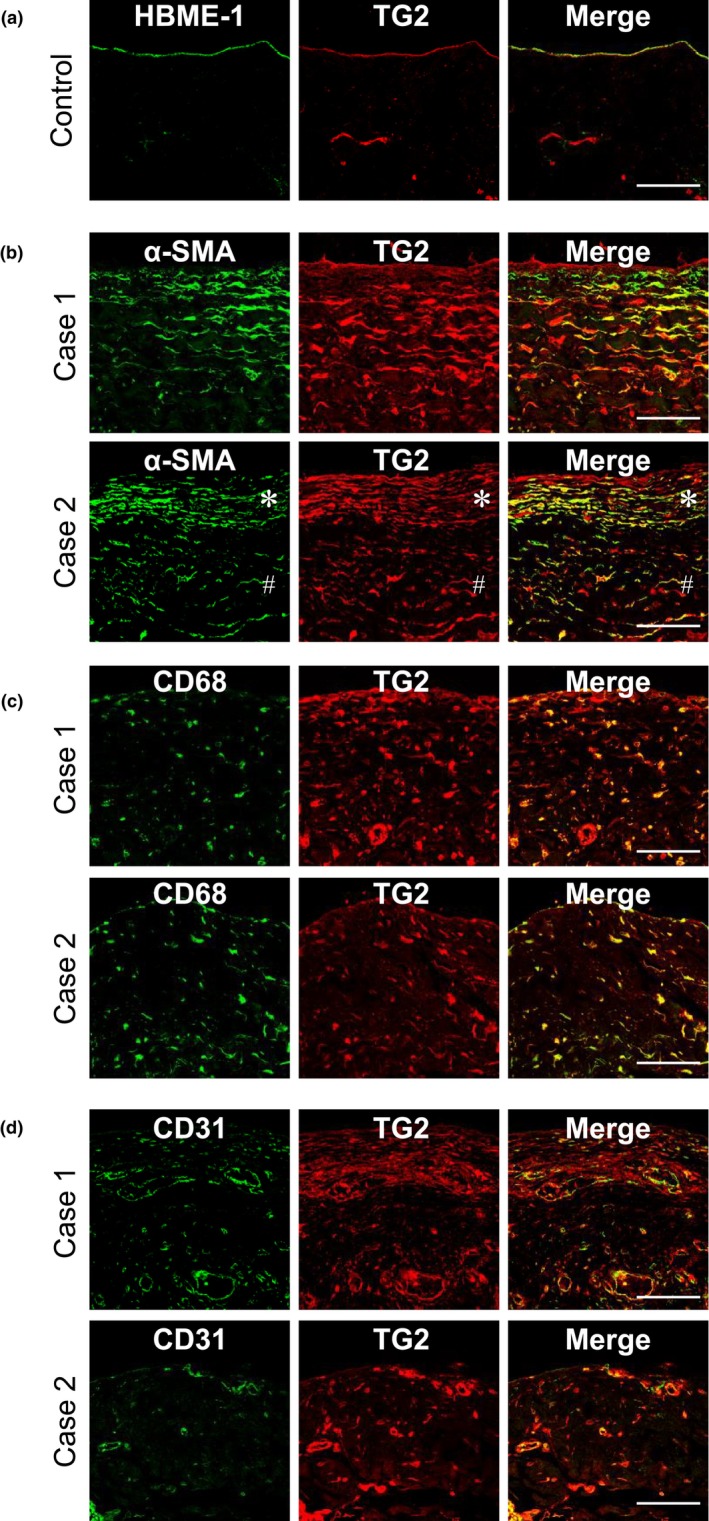
Double immunostaining for TG2 and markers of mesothelial cells, myofibroblasts, macrophages, and vascular endothelial cells in the human peritoneal membrane. Representative images of double staining for TG and markers of mesothelial cells, myofibroblasts, macrophages, and vascular endothelial cells. (a) In the control peritoneum, TG2 (red) was positive in mesothelial cells (green), as detected by the anti‐HBME antibody. (b) TG2 (red) is expressed by α‐SMA‐positive myofibroblasts (green). Case 1: Peritoneum of a patient with bacterial peritonitis. Case 2: Peritoneum of a patient with EPS. TG2 is expressed by α‐SMA‐positive myofibroblasts in the original peritoneal membrane (#) and newly synthesized encapsulating membrane (*). (c) TG2 (red) is expressed by CD68‐positive macrophages (green). Case 1: Peritoneum of a patient with bacterial peritonitis. Case 2: Peritoneum of a patient treated with conventional acidic solutions. (d) TG2 (red) is expressed by CD31‐positive endothelial cells (green). Case 1: Peritoneum of a patient with bacterial peritonitis. Case 2: Peritoneum of a patient treated with a low‐GDP pH‐neutral solution who frequently experienced episodes of peritonitis (0.82 episodes/patient‐year). HE staining results and the findings for TG2, α‐SMA, and CD68 expression are shown in Figure S1. α‐SMA, α‐smooth muscle actin; anti‐HBME antibody, Monoclonal anti‐mesothelial cell antibody; EPS, encapsulating peritoneal sclerosis; TG2, Transglutaminase 2. Scale bars = 100 μm.

Double staining was performed to identify the cells expressing TG2 in the human peritoneum. In the control peritoneum, TG2 colocalized with mesothelial cells (Figure 4a). TG2 was expressed by α‐SMA‐positive myofibroblasts in the original and newly synthesized encapsulating membrane (Figures 2b and 4b) and by CD68‐positive macrophages (Figure 4c). TG2 is involved in the progression of endothelial‐to‐mesenchymal transition (EndMT) (Fell et al., 2021; Kunoki et al., 2023). TG2 was often expressed by endothelial cells in cases of severe peritonitis (Case 1 in Figure 4d) or peritoneal fibrosis after frequent episodes of peritonitis, even when a low‐GDP pH‐neutral solution was used (Case 2 in Figure 4d and Figure S1). These findings were consistent with those obtained using the CG‐induced inflammation and fibrosis model (Kunoki et al., 2023). In contrast, CD31 and TG2 were not detected in vessels with severe vasculopathy in EPS cases, likely because of the loss of antigenicity (Figure S2).

### 3.3 Interaction between TG2 and TGF‐β in mesothelial cells

**FIGURE 5:**
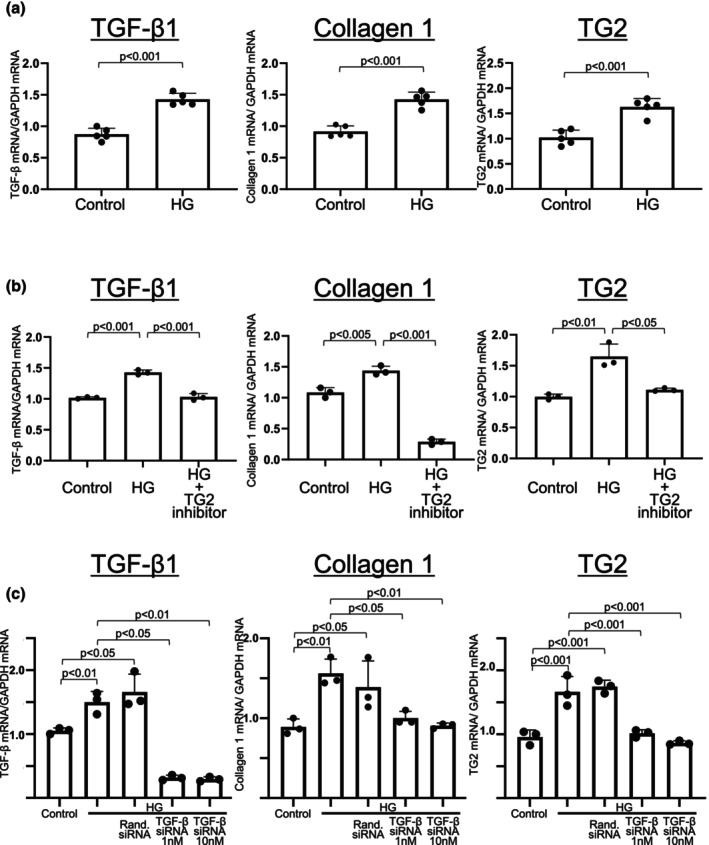
Interaction between TG2 and TGF‐β expression in cultured mesothelial cells. (a) High‐glucose condition (90 mM glucose concentration) induced upregulation of TGF‐β1, collagen 1, and TG2 expression. (b) High‐glucose condition‐induced upregulation of TGF‐β1, collagen 1, and TG2 was suppressed by the addition of a TG2 inhibitor. (c) High‐glucose condition‐induced upregulation of TGF‐β1, collagen 1, and TG2 was suppressed by TGF‐β1 siRNA, but not by random siRNA. Control, culture medium containing 5 mM glucose; HG, High glucose (culture medium containing 90 mM glucose); Rand. siRNA, Random small interfering RNA; TG2, transglutaminase 2.

Mesothelial cells have been reported to play an important role in the development of peritoneal fibrosis caused by high glucose exposure, mainly through the TGF‐β/Smad signaling pathway, but not by equimolar concentrations of d‐mannitol (Devuyst et al., 2010; Ito et al., 2024; Jiang et al., 2020; Kang et al., 1999). We investigated the interaction between TG2 and TGF‐β in mesothelial cells. A high glucose concentration of 90 mM induced the upregulation of TGF‐β1, collagen 1, and TG2 in Met‐5A mesothelial cells (Figure 5a). High‐glucose–induced upregulation of TGF‐β1 and collagen 1 was suppressed by a TG2 inhibitor (Figure 5b). In addition, high‐glucose–induced upregulation of TGF‐β1, collagen 1, and TG2 was suppressed by TGF‐β1 siRNA but not by control siRNA (Figure 5c).

## 4 DISCUSSION

In this study, we analyzed the expression of TG2 in 163 human peritoneal membrane tissue samples obtained at the time of catheter removal or surgical enterolysis from patients with PD, including those with peritonitis and EPS. To our knowledge, this is the first report describing the TG2 expression pattern in human peritoneal fibrosis. We clearly showed that TG2 was upregulated in the thickened peritoneum, and this upregulation was associated with strong expression of α‐SMA and CD68‐positive macrophages (Figures 2 and 3). These findings were similar to those for previously reported CG‐induced peritoneal fibrosis models (Kunoki et al., 2023). TG2 is expressed by myofibroblasts, macrophages, and vascular endothelial cells (Figure 4), as shown in animal models and previous in vitro studies (Kunoki et al., 2023). TG2 is involved in epithelial‐mesenchymal transition (EMT) and EndMT (Fell et al., 2021; Soltani & Kaartinen, 2023; Tatsukawa et al., 2018; Wang et al., 2020). The increased expression of type 1 collagen, α‐SMA, TG2, TGF‐β, CD68, CD31, and VEGF‐A in the CG model was significantly downregulated in TG2‐knockout mice (Kunoki et al., 2023). In this human study, TG2 expression was the highest in patients with peritonitis (Figures 2a and 3a). In addition, strong TG2 expression was observed in endothelial cells, myofibroblasts, and macrophages (Figure 4b–d), consistent with the phenomena observed in the CG model of peritonitis‐induced peritoneal fibrosis (Kunoki et al., 2023). Interleukin (IL)‐6 and tumor necrosis factor (TNF)‐α, which are known to be upregulated in peritonitis, may contribute to the induction of TG2 not only in fibroblasts but also in endothelial cells (Bhedi et al., 2020; Luo et al., 2016), potentially through the Janus kinase 2 (JAK2)/signal transducer and activator of transcription 3 (STAT3) or nuclear factor kappa‐light‐chain‐enhancer of activated B cells (NF‐κB) signaling pathway (Liu et al., 2024; Luo et al., 2016).

The characteristic pathological features of EPS include encapsulation of a new membrane with peritoneal fibrosis, fibrin deposition, swollen fibroblasts, capillary angiogenesis, and inflammatory cell infiltration (Alston et al., 2017; Honda et al., 2017; Honda & Oda, 2005; Ito et al., 2024). The early loss of sodium sieving has been reported to be a predictor of EPS (Barreto et al., 2019; Morelle et al., 2015). This loss of sodium sieving has been reported to be due to a large increase in collagen density (Morelle et al., 2015). Therefore, high collagen density is a risk factor for the development of EPS. Upregulation of TG2 expression was also observed in the EPS group in this study, which may enhance the cross‐linking of collagen through TGF‐β activity. Based on clinical and pathophysiological conditions, EPS is classified into four stages: pre‐EPS, inflammatory, encapsulating, and chronic stages (Al‐Lawati et al., 2020; Ito et al., 2024; Nakayama et al., 2023). In the earlier stages, the pathophysiology tends to be more inflammatory with minimal sclerosis. In the later stages, the pathophysiology tends to be more sclerotic with less inflammation. In the advanced sclerotic stage, patients may present with partial or complete bowel obstruction (Al‐Lawati et al., 2020; Ito et al., 2024). The peritoneal tissues of patients with EPS in the present study tended to be sclerotic and less inflammatory (Case 1 in Figure 2b). This is one of the reasons why the number of CD68‐positive macrophages in the peritoneal tissues of the EPS cases was lower than that in the peritonitis cases, despite the high peritoneal thickness (Figure 3a). However, in some cases, such as Case 2 in Figure 2b, inflammatory changes were observed. Therefore, the number of CD68‐positive cells and the α‐SMA‐positive area are widely distributed in Figure 3a. TG2 expression was correlated with inflammation (macrophage infiltration) and, to a lesser extent, with the fibrotic process (α‐SMA expression) even in the chronic stage of EPS cases (Figure S3). TG2 was highly expressed not only in the original peritoneal membrane, but also in the newly synthesized encapsulating membrane (Figures 2b and 4b), which may have contributed to the high density of collagen in the original peritoneum and strong adhesion between the intestines.

To date, the interaction between TG2 and TGF‐β has mainly been demonstrated in fibroblasts in the peritoneum. In fibroblasts, TGF‐β1 induces TG2 (Fell et al., 2021; Quan et al., 2005; Ritter & Davies, 1998), whereas TG2 upregulates TGF‐β1 (Telci et al., 2009). In fibroblasts, there is a positive feedback effect of TG2 on TGF‐β1 which promotes further fibrosis (Quan et al., 2005; Ritter & Davies, 1998; Telci et al., 2009). Suppression of TG2 downregulates TGF‐β1 expression (Telci et al., 2009; Wang et al., 2017). Additionally, high‐glucose conditions upregulate TG2 as well as TGF‐β1, which is blocked by a TG2 inhibitor (Bhedi et al., 2020). However, these phenomena were observed in fibroblasts, but not in mesothelial cells. In this study, we clearly demonstrated the same interactive regulation between TGF‐β1 and TG2 expression in mesothelial cells. The upregulation of TG2 and TGF‐β1 was suppressed by TGF‐β1 siRNA and TG2 inhibitor, respectively (Figure 5). These results suggest that high glucose levels in the PD solution induce peritoneal fibrosis, even when low‐GDP pH‐neutral solutions are used instead of conventional acidic solutions. Therefore, the development of a dialysate using osmotic agents other than glucose is required.

Recent advances in the development of pharmacological TG2 inhibitors have provided valuable tools for investigating the role of TG2 in fibrosis and inflammation. ZED1227, a selective and orally active TG2 inhibitor, has entered clinical trials for celiac disease and demonstrated favorable pharmacokinetics, target engagement, and tolerability in humans (Schuppan et al., 2021). In preclinical studies, ERW1041E, a dihydroisoxazole‐based compound, has been utilized as a TG2 inhibitor and shown efficacy in reducing fibrosis and cardiac dysfunction in models of pressure overload (Shinde et al., 2018). In addition to these TG2‐targeted compounds, several medications not originally designed to inhibit TG2 have been reported to exert TG2‐inhibitory effects as off‐target actions. For example, cysteamine and its oxidized form, cystamine, competitively inhibit TG2 and have been shown to reduce fibrosis in hepatic and renal models (Qiu et al., 2007). Furthermore, PPARγ agonists such as pioglitazone and 15‐deoxy‐Δ^12^, ^14^‐prostaglandin J₂ have demonstrated antifibrotic effects that may partly involve indirect suppression of TG2 expression or activity, although TG2 is not their primary target (Burgess et al., 2005). While these off‐target effects have not yet been evaluated in peritoneal fibrosis, they underscore the broader pharmacological potential of modulating TG2 activity.

This study had several limitations. First, all patients enrolled in this study were Japanese; therefore, the generalizability of these findings to other populations requires careful consideration, since the occurrence rate of EPS differs among countries. Second, we could not evaluate the effects of PD duration, extent of glucose exposure, icodextrin use, dialysis failure incidence, or EPS stage on TG2 expression. Additionally, we did not evaluate the effects of steroid treatment on TG2 expression in patients with EPS. Third, extrapolating these data to children may be challenging because responses to PD solutions may differ between adults and children (Schaefer et al., 2018). Peritoneal thickness and blood capillary density change during growth (Schaefer et al., 2016), which may influence the different responses to stimuli in adults and children.

Fourth, the conventional PD acidic solution was changed to a pH‐neutral solution in 2005. Consequently, peritoneal tissues from the conventional and pH‐neutral solution eras were not collected in the same period. These differences may reflect changes in clinical practice that affect TG2 expression. Fifth, in Japan, prolonged PD treatment with conventional PD solutions resulted in a high incidence of EPS. After switching to pH‐neutral, low‐GDP solutions and shortening treatment duration, incidence rates decreased to 1.0% (Nakayama et al., 2023). In this EPS cohort, the incidence rate was not evaluated because of the limited number of patients who were referred to Tsuchiya General Hospital for enterolysis surgery.

In conclusion, this is the first study to investigate the expression and role of TG2 in human peritoneal fibrosis and EPS. The findings indicate that TG2 is involved in peritoneal injury during PD. A high‐glucose dialysate induces peritoneal fibrosis through the interaction between TGF‐β and TG2 in cultured mesothelial cells. The findings of this study, together with our previous animal studies (Kunoki et al., 2023), suggest that TG2 is a potential therapeutic target for preventing peritoneal injury and EPS in PD.

## AUTHOR CONTRIBUTIONS

H.Ki., K.H., Y.S., T.I., and Y.I. designed the study. H.Ki., K.K., H.T., M.S., M.T., H.S., Y.M., H.Ki., and M.Ik. performed the experiments. M.Y., M.M., M.B., K.T., H.Ki., and H.Ka. collected the human tissues and analyzed the data. M.Iw., T.I., K.T., and Y.I. prepared the figures and drafted the manuscript. All authors have approved the final manuscript.

## FUNDING INFORMATION

Part of this work was supported by a Grant‐in‐Aid for Scientific Research from the Ministry of Education, Science, and Culture, Japan (Government of Japan, Y.I., grant number 24K11421), Japanese Association of Dialysis Physicians (Y.I. grant number 2022‐6) and a research grant from the Tsuchiya Memorial Medical Foundation (Y.I. grant number 2022‐1).

## CONFLICT OF INTEREST STATEMENT

The authors declare no conflicts of interest.

## ETHICS STATEMENT

This study was approved by the Ethics Committee for Human Research of the Faculty of Medicine at Nagoya University (approval number: 299‐7).

## CONSENT

Informed consent was obtained from all the patients.

## Supporting information


**Figure S1:** Expression of TG2, α‐SMA, and CD68 in the case treated with a low‐GDP pH‐neutral solution who frequently experienced episodes of peritonitis. Same patient as Case 2 in Figure 4d. The peritoneum was obtained from a patient treated with a low‐GDP pH‐neutral solution who frequently experienced episodes of peritonitis (0.82 episodes/patient‐year). Increased TG2 expression was associated with peritoneal fibrosis. TG2 was expressed in the blood vessels and colocalized with CD31 (Case 2 in Figure 4d). The figures on the right show an enlarged view of the square on the left. Scale bar = 100 μm.
**Figure S2:** Severe vasculopathy in the case with advanced sclerotic EPS. The patient had bowel obstruction due to an advanced sclerotic stage of EPS. Vessels with severe vasculopathy (arrows) expressed neither CD31 nor TG2. The picture at the bottom left is an enlarged version of the one at the top right. Scale bars = 100 μm.
**Table S1:** List of primers used for real‐time PCR (TaqMan gene expression assay).
**Table S2:** Company and catalogue numbers of the reagents.

## Data Availability

The data underlying this article will be shared on reasonable request to the corresponding author.
