# Supplementary material for: Transglutaminase 2 in human peritoneal dialysis‐related peritoneal injury
Source: Physiol Rep. 2025 Sep 22;13(18):e70567. doi: 10.14814/phy2.70567 (PMC12451402; doi:10.14814/phy2.70567)
Supplement: Supplementary file 1 — Figure S1: Expression of TG2, α‐SMA, and CD68 in the case treated with a low‐GDP pH‐neutral solution who frequently experienced episodes of peritonitis. Same patient as Case 2 in Figure 4d. The peritoneum was obtained from a patient treated with a low‐GDP pH‐neutral solution who frequently experienced episodes of peritonitis (0.82 episodes/patient‐year). Increased TG2 expression was associated with peritoneal fibrosis. TG2 was expressed in the blood vessels and colocalized with CD31 (Case 2 in Figure 4d). The figures on the right show an enlarged view of the square on the left. Scale bar = 100 μm. Figure S2: Severe vasculopathy in the case with advanced sclerotic EPS. The patient had bowel obstruction due to an advanced sclerotic stage of EPS. Vessels with severe vasculopathy (arrows) expressed neither CD31 nor TG2. The picture at the bottom left is an enlarged version of the one at the top right. Scale bars = 100 μm. Table S1: List of primers used for real‐time PCR (TaqMan gene expression assay). Table S2: Company and catalogue numbers of the reagents. [file PHY2-13-e70567-s001.pdf]

Supplemental Materials

- Title: Transglutaminase 2 in human peritoneal dialysis-related peritoneal injury
-
- Authors: Shunnosuke Kunoki, Masashi Ikeno, Hideki Tatsukawa, Yukinao Sakai, Hiroshi Kinashi, Keisuke Kamiya, Masafumi Suzuki, Masashi Mizuno, Makoto Yamaguchi, Hiroyuki Sasakura, Yuki Morioka, Masataka Banshodani, Mitsuhiro Tawada, Masato Iwabu, Takuji Ishimoto, Kosei Takeuchi, Kiyotaka Hitomi, Hideki Kawanishi, and Yasuhiko Ito

- **Supplementary Table 1**: List of primers used for real-time PCR (TaqMan gene expression assay)
- **Supplementary Table 2. Company and catalogue numbers of the reagents**
- **Supplementary Figure 1**: . Expression of TG2, α-SMA, and CD68 in the case with EPS.
- **Supplementary Figure 2**: Severe vasculopathy in the case with advanced sclerotic EPS.

Supplementary Tables

Supplementary Table 1. List of primers used for real-time PCR (TaqMan gene expression assay)

	Assay identification number
human type I collagen	Hs00164004_m1
human TGF-β1	Hs00171257_m1
human TGM2	Hs01096681_m1
human GAPD (GAPDH)	4333764F
18S ribosomal RNA	Hs99999901_s1

Supplementary Table 2. Company and catalogue numbers of the reagents

Reagents	Catalogue number	Company
DAB Substrate Kit	425011	Nichirei Bioscience, Tokyo, Japan
BSA	011-15144	FUJIFILM, Tokyo, Japan
siRNA Universal Negative Control	SIC002	Sigma-Aldrich, St.Louis, MO
Lipofectamine™ RNAiMAX Transfection Reagent	13778030	Thermo Fisher Scientific, Waltham, MA
Boc-DON-Gln-Ile-Val-OMe	B003	Zedira GmbH, Darmstadt, Germany
Rneasy Lipid Tissue Mini Kit	74804	QIAGEN, Hulsterweg, Netherlands
TaqMan Fast Advanced Master Mix	4444963	Thermo Fisher Scientific, Waltham, MA
QuantiTect Reverse Transcription Kit	205313	QIAGEN, Hulsterweg, Netherlands

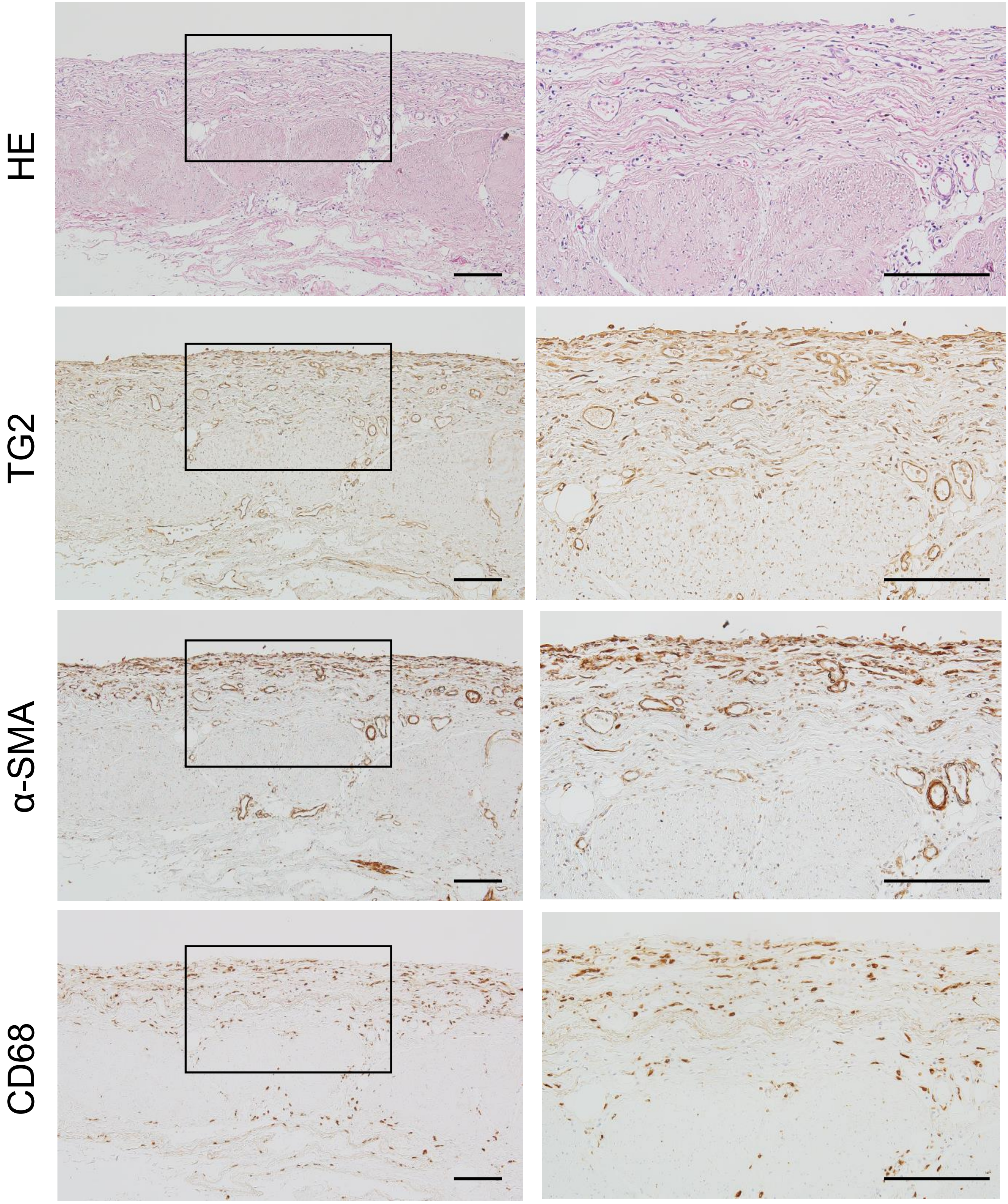

Supplementary Figure 1. Expression of TG2, α-SMA, and CD68 in the case treated with a low-GDP pH-neutral solution who frequently experienced episodes of peritonitis.

Same patient as Case 2 in Figure 4D

The peritoneum was obtained from a patient treated with a low-GDP pH-neutral solution who frequently experienced episodes of peritonitis (0.82 episodes/patient-year). Increased TG2 expression was associated with peritoneal fibrosis. TG2 was expressed in the blood vessels and colocalized with CD31 (Case 2 in **Figure 4D**).

The figures on the right show an enlarged view of the square on the left. Scale bar = 100 μm

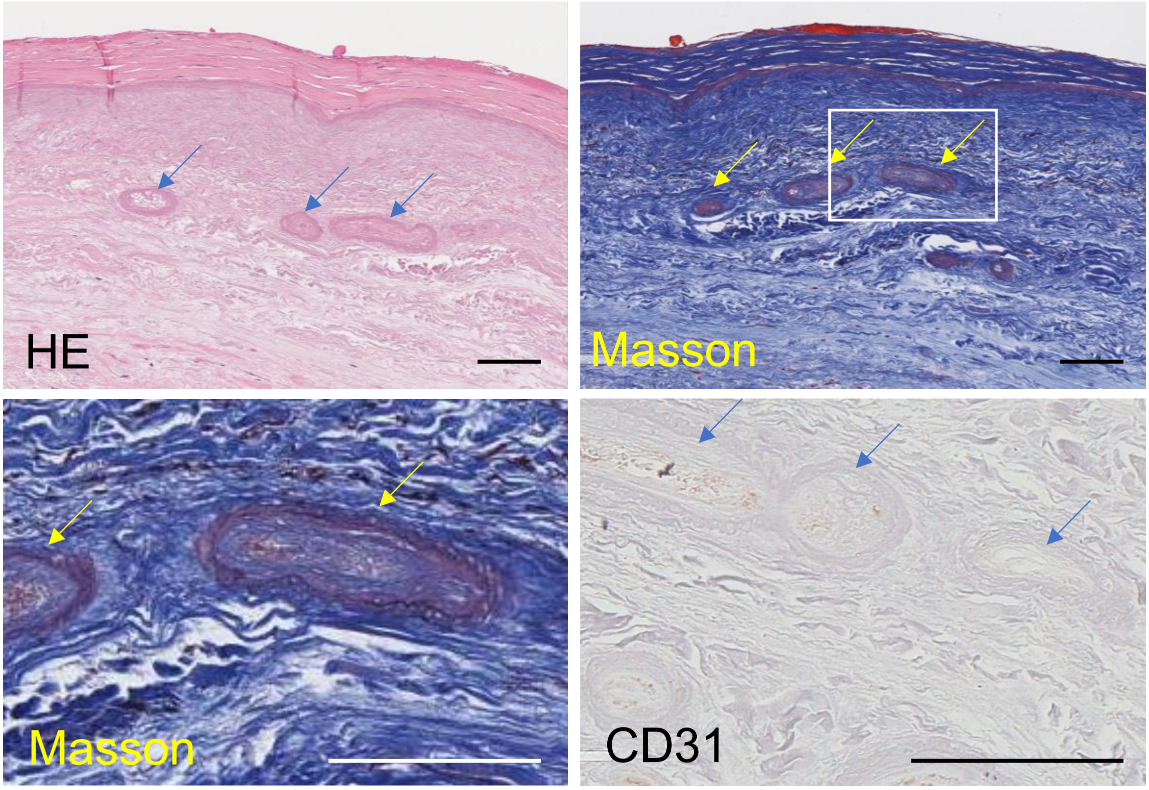

Supplementary Figure 2. Severe vasculopathy in the case with advanced sclerotic EPS. The patient had bowel obstruction due to an advanced sclerotic stage of EPS. Vessels with severe vasculopathy (arrows) expressed neither CD31 nor TG2. The picture at the bottom left is an enlarged version of the one at the top right.
Scale bars = 100 μm

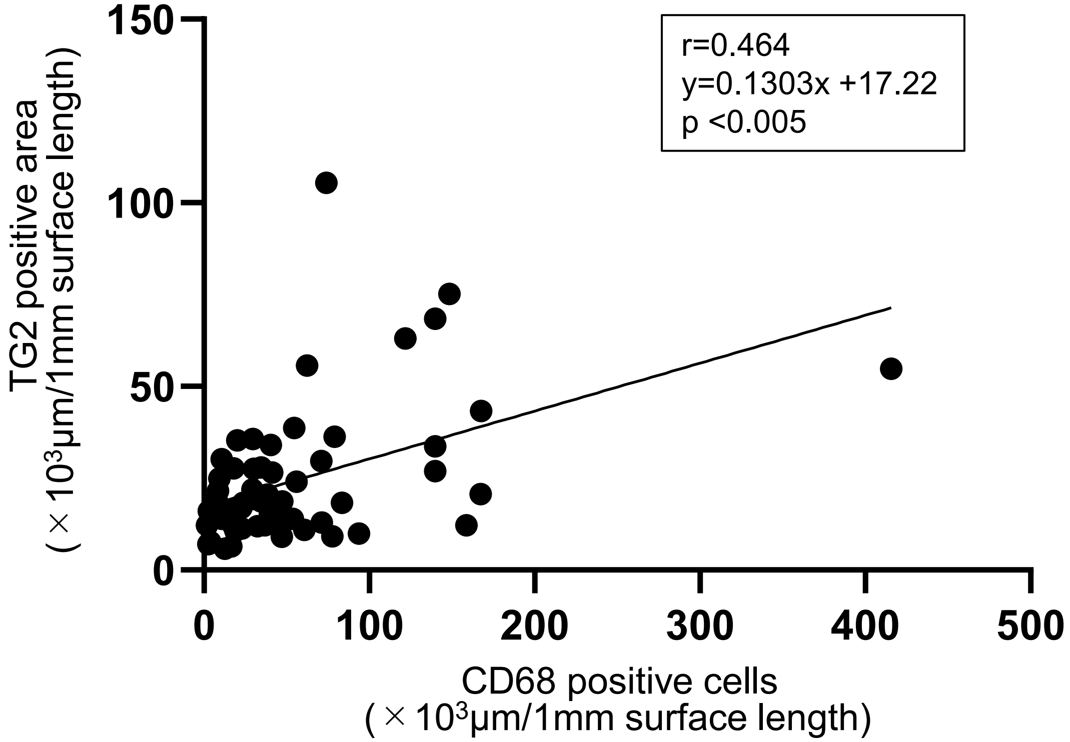

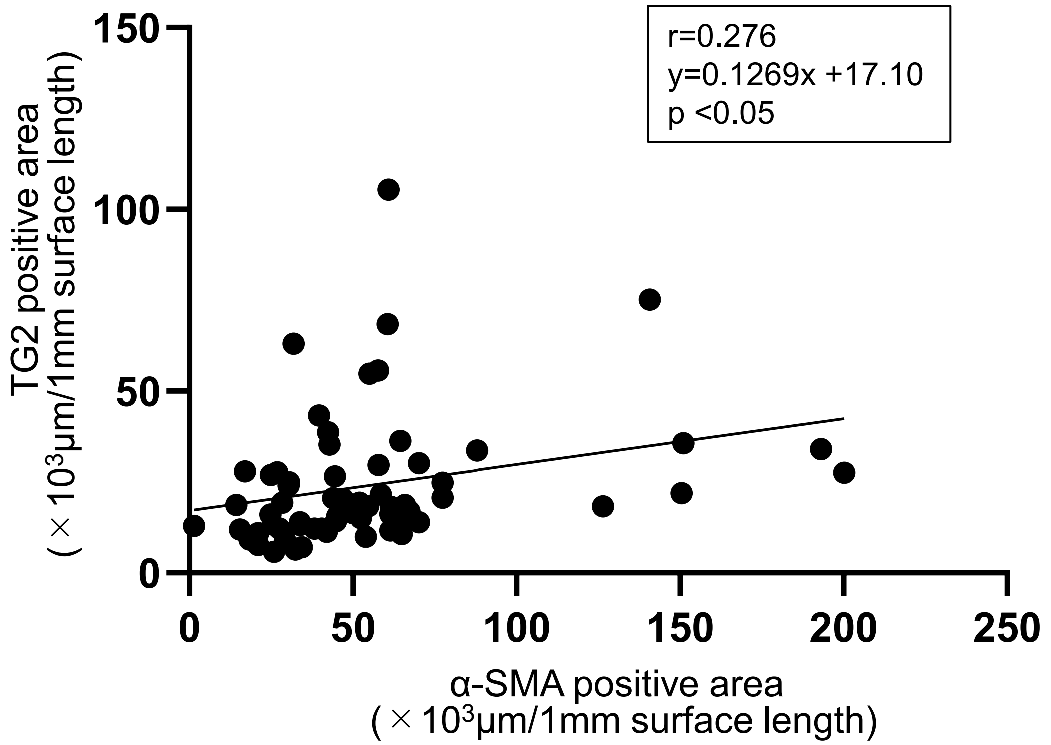

Supplementary Figure 3. The relationship between TG2 expression and inflammation or the fibrotic process in EPS cases.

TG2 expression was correlated with inflammation (macrophage infiltration) and, to a lesser extent, with the fibrotic process (α-SMA expression) in EPS cases.
